# Walshe on Diseases of the Lungs

**Published:** 1860-09

**Authors:** 


WALSHE ON DISEASES OF THE LUNGS.

Walter Hayde Walshe, M. D., is already favorably known to the Medical profession of the United States, as well as of Great Britain, as an eminent authority on diseases of the lungs and appendages, and the present edition of his work, from the third revised and enlarged London edition, will be found a valuable accession to this branch of Pathological literature.—The work before us is much fuller and more complete than former editions; containing, besides more elaborate details of diagnosis, prognosis and treatment—points of especial interest to the student—considerations of diseases not previously mentioned.—His style is forcible, clear and concise; the instructions for inspections, and the descriptions of the various phenomena of sounds, murmurs, signs, &c., precise, graphic and unmistakable. We are tempted, however, to observe that the section on *Change of Climate* might have been profitably omitted by the American Publishers; ignoring, as it does, almost totally the existence of the North American Continent, with its almost endless variety of climatic and atmospheric conditions.

Too little attention has as yet been paid by the European, but more especially the British practititioner, to the advantages offered, even within the boundaries of the United States, for climatic treatment—advantages pre-eminent in possessing kindred customs and language, and the comforts, usages and appliances of civilization.

This, however, is of minor importance as affecting the value of the work in this country; while for exhaustive treatment of subject, soundness and reliability of dicta, and eminently readable style, terse yet lucid, it merits our hearty approval.

